# An Exploratory Pilot Study of Changes in Global DNA Methylation in Patients Undergoing Major Breast Surgery Under Opioid-Based General Anesthesia

**DOI:** 10.3389/fphar.2021.733577

**Published:** 2021-09-21

**Authors:** Francesca Felicia Caputi, Lucia Carboni, Laura Rullo, Irene Alessandrini, Eleonora Balzani, Rita Maria Melotti, Patrizia Romualdi, Sanzio Candeletti, Andrea Fanelli

**Affiliations:** ^1^ Department of Pharmacy and Biotechnology, Alma Mater Studiorum—University of Bologna, Bologna, Italy; ^2^ Department of Surgical Sciences, University of Turin, Turin, Italy; ^3^ Department of Surgical and Medical Sciences, Alma Mater Studiorum—University of Bologna, Bologna, Italy; ^4^ Anesthesiology and Pain Therapy Unit, AOSP S.Orsola Hospital, Bologna, Italy

**Keywords:** pain, opioids, anesthesia, DNA methylation, gene expression, DNA methyltransferases, cytokines, surgery

## Abstract

This study aimed to investigate DNA methylation levels in patients undergoing major breast surgery under opioid-based general anesthesia. Blood samples were collected from eleven enrolled patients, before, during and after anesthesia. PBMC were isolated and global DNA methylation levels as well as DNA methyltransferase (DNMT) and cytokine gene expression were assessed. DNA methylation levels significantly declined by 26%, reversing the direction after the end of surgery. Likewise, DNMT1a mRNA expression was significantly reduced at all time points, with lowest level of −68%. DNMT3a and DNMT3b decreased by 65 and 71%, respectively. Inflammatory cytokines IL6 and TNFα mRNA levels showed a trend for increased expression at early time-points to end with a significant decrease at 48 h after surgery. This exploratory study revealed for the first time intraoperative global DNA hypomethylation in patients undergoing major breast surgery under general anesthesia with fentanyl. The alterations of global DNA methylation here observed seem to be in agreement with DNMTs gene expression changes. Furthermore, based on perioperative variations of IL6 and TNFα gene expression, we hypothesize that DNA hypomethylation may occur as a response to surgical stress rather than to opiate exposure**.**

## Introduction

Epigenetic alterations modulate gene expression by changes in chromatin structure mainly brought about by covalent modifications of histone proteins and by DNA methylation (Bernstein et al., 2007). DNA methylation is one of the epigenetic mechanisms contributing to postoperative hyperalgesia development (Sun et al., 2015).

Opioid-induced hyperalgesia (OIH) is a hypersensitivity to painful stimuli that can develop after exposure to opioid analgesics (Bannister and Dickenson, 2010). It occurs in opioid treatment for acute or chronic pain causing an unexplained paradoxical pain sensitivity (Roeckel et al., 2016). Perioperative opioid administration seems to play an important environmental role in causing OIH (Guignard et al., 2000; van Gulik et al., 2012; Colvin et al., 2019). Cellular and molecular mechanisms sustaining OIH (Bannister and Dickenson, 2010; Roeckel et al., 2016; Colvin et al., 2019) are not completely understood. A possible role for epigenetic mechanisms in an OIH context has been deemed, based on epigenetic changes underpinning chronic pain conditions and on suggestive preclinical data (Doehring et al., 2011; Liang et al., 2013; Liang et al., 2015; Ligon et al., 2016).

DNA methylation consists of a methyl group addition on carbon 5 in a cytosine base to create 5-methylcytosine (5-mC) in promoter or enhancer regions, and it usually occurs on cytosine-phosphate-guanine dinucleotide sequences (Jaenisch and Bird, 2003). It consists of a gene silencing mechanism, regulated by DNA methyltransferases (DNMTs). When DNMT activity is suppressed, DNA passive demethylation takes place, resulting in unmethylated DNA. In mammals, three forms of active DNMTs (DNMT1, DNMT3a, and DNMT3b) are known (Jurkowska et al., 2011).

Available evidence supports the role exerted by DNMTs in pain-induced molecular alterations. In rodent models of neuropathic pain and nerve injury, DNMT3s are considerably and persistently up-regulated in dorsal root ganglia (DRG), and spinal cord (Pollema-Mays et al., 2014; Shao et al., 2017). DNMT1 is upregulated in dorsal root ganglia after peripheral nerve injury (Sun et al., 2019). The DNMT3a and DNMT3b isoforms’ expression levels are increased in inflammatory and chemotherapy-induced pain models (Abzianidze et al., 2014; Mao et al., 2019), suggesting their involvement in different pain conditions.

Furthermore, previous reports described a connection between opioid exposure and DNA hypermethylation on the µ opioid receptor promoter (Nielsen et al., 2009; Chorbov et al., 2011; Wachman et al., 2014; Wachman et al., 2018).

Despite limited available data, opiates constitute a heterogeneous group of drugs widely used perioperatively whose epigenetic effects have not been investigated. Fentanyl is the most commonly used opioid drug to ensure analgesia in general anesthesia. Fentanyl ability to induce OIH has been demonstrated in clinical studies and in pre-clinical models (Waxman et al., 2009; Yildirim et al., 2014; Lyons et al., 2015; Mauermann et al., 2016; Araldi et al., 2018; Rupniewska-Ladyko and Malec-Milewska, 2019). A number of molecular modifications have been associated to OIH occurrence, including nociceptor neuroplasticity induction, µ receptor signaling pathway alteration, increased expression of proinflammatory cytokines, COX-2 mRNA and spinal PGE2 variations, as well as DNA methylation (Doehring et al., 2013; Koblish et al., 2017; Li et al., 2017; Chang et al., 2018; Li et al., 2018; Zeng et al., 2018; Khomula et al., 2019; Zhang et al., 2019).

Based on these evidences, the present study aimed to investigate DNA methylation variations, as well as DNMTs, IL6 and TNFα gene expression in peripheral blood mononuclear cells (PBMC) of patients undergoing major surgery which were intraoperatively exposed to fentanyl according to current clinical practice. PBMC are the usual blood cells chosen to investigate proteins and nucleic acids in humans and they are useful as a model of epigenetic gene regulation in the brain (Gavin and Sharma, 2010).

## Materials and Methods

### Study Design

The present study is an observational retrospective trial conducted in line with the Declaration of Helsinki on Biomedical Research Involving Human Subjects and reported according to the STROBE (STrengthening the Reporting of OBservational studies in Epidemiology) checklist (von Elm et al., 2014). Approval from the ethics committee of the Sant’Orsola-Malpighi Hospital of Bologna (practice number 0012809) was obtained and the trial was registered on clinicaltrials.gov (NCT02938455). A written informed consent was obtained by each participating subject. The participation to the study did not interfere with the normal treatment for each patient, as the therapeutic plan represented normal daily clinical practice and it was carried out regardless of patient enrollment. Given the explorative nature of the study, we decided initially to enroll 20 patients. Following evaluations that emerged from an interim analysis, in which the results appeared to be in contrast with the primary hypothesis, it was agreed to stop enrollment. Based on the potential alterations which characterize the perioperative stress response it was therefore deemed appropriate to expand the analysis to inflammatory cytokine expression to better interpret the results and properly plan future research on this topic.

### Study Population

Patients undergoing major surgery performed under general anesthesia, referring to the Anesthesiology unit of Policlinico di Sant’Orsola were enrolled to the present study.

The inclusion criteria enrolled individuals of both sexes who were aged 18–65 years and had body mass index (BMI in kg/m^2^) from 18 to 35, with anesthetic risk (American Society of Anesthesiologists physical status classification system-ASA) ≤ 3 and listed for elective major surgery of expected duration longer than 120 min performed under general anesthesia, according to daily clinical practice. Exclusion criteria were: severe renal and hepatic failure, a previous diagnosis of obstructive sleep apnea, opioid intake within 30 days before surgical procedure and an Apfel score for the evaluation of post-operative nausea and vomiting higher than 3.

We collected anthropometric parameters (sex, age, height, weight, BMI), duration of anesthesia (time between induction of general anesthesia and awakening), duration of surgery (time between surgical incision and the last stitch), fentanyl intraoperative consumption (mcg/kg/hour), and, if available from patient record, morphine consumption at 24 and at 48 h postoperatively, Number Rating Scale (NRS) at rest, NRS after deep inhalation 10 min after waking up, at 24 and at 48 h postoperatively, number of episodes of nausea and vomiting at 24 and at 48 h postoperatively, and consumption of antiemetic drugs at 24 and at 48 h postoperatively.

For subsequent molecular analyses, each patient was assigned a progressive number in order to anonymize data.

### Anesthesia and Surgery

Standard therapeutic plans were followed irrespective of patient’s enrollment. Patients were monitored in relation to the type of surgery (ECG, SpO_2_, EtCO_2_ and blood pressure). After monitoring the patient, a peripheral venous catheter ≤20 G and an arterial catheter (20 G) were placed in a radial or brachial artery.

In our clinical practice, after adequate oxygenation anesthesia was usually induced with fentanyl (3 mcg/kg), propofol (2–2.5 mg/kg) and rocuronium (0.6 mg/kg) or succinylcholine (1 mg/kg). The maintenance of anesthetic level was obtained using sevoflurane (0.7–1.3 Minimum Alveolar Concentration), and bolus doses of fentanyl (1 mcg/kg). Rocuronium (0.15 mg/kg) was administered every 45 min.

A bolus of morphine (usually 0.1 mg/kg) was administered 40 min before awakening. If pain was poorly controlled (NRS ≥4) ten minutes after the awakening, 1 mg of morphine was administered. The bolus could be repeated every 5 min, until NRS resulted less than 3. Acute postoperative pain was treated in the first 48 h with a continuous intravenous infusion of morphine (1 mg/h). The patient could also self-administer 1 mg morphine bolus every 8 min.

Prophylaxis for postoperative nausea and vomiting (PONV) was carried out by intravenous administration of 0.1 mg/kg of dexamethasone prior to administration of the first dose of fentanyl. In case of postoperative nausea and vomiting, droperidol (0.625 mg) was given every 6 h.

### Blood Sample Collection

In each patient, five peripheral blood samples were collected for subsequent molecular analyses according to the following schedule: • T0: after arterial catheter placement, before opiate administration (basal blood gas analysis); • T1: 1 h after the induction of general anesthesia (blood gas analysis verifying mechanical ventilation); • T2: after the last stitch, before the intraoperative morphine bolus (blood gas analysis to check blood loss); • T3: 24 ± 6 h after the last stitch (complete blood count); • T4: 48 ± 6 h after the last stitch (complete blood count).

Blood samples were added with heparin or EDTA or ACD ascorbate as anticoagulant agents and stored at a temperature of +4°C until analysis.

### Isolation of Mononuclear Cells From Peripheral Blood

Peripheral blood mononuclear cells (PBMCs) were isolated by standard Ficoll–hystopaque density gradient centrifugation. PBMCs were isolated from whole blood samples of each participant collected from T0 to T4. For this purpose, fresh blood (about 6 ml) was diluted 1: 1 with PBS (Dulbecco’s Phosphate Buffered Saline, cat. # BE17-516F, Lonza, Basel, Switzerland), and gently layered on top of Lympholyte (Cell Separation Media Human, cat. # CL5015, Cedarlane Laboratories**,** Burlington, Canada) to keep blood and Lympholyte as two different layers. Tubes were then centrifuged at 800 × g for 20 min at room temperature (with centrifuge brakes off). At the end of the centrifugation the mononuclear cells stratified in the Lympholyte-plasma interface were collected. PBMCs were then washed three times with 10 ml of sterile PBS at 300 × g for 8 min (with the centrifuge brakes ON).

### DNA and RNA Extraction

Freshly isolated PBMCs were subsequently subjected to nucleic acids extraction. DNA and RNA were extracted by the ZR-Duet™ DNA/RNA MiniPrep (cat. #D7001, Zymo Research, Orange, CA, United States) which provides a quick method for the isolation of high-quality genomic DNA and total RNA. According to the manufacturer’s instructions, PBMC cells were suspended and directly processed by adding 400 μL of DNA/RNA lysis buffer, and then transferred into a Zymo-Spin IIIC Column to be centrifuged at 12.000 × g for 1 min. The flow-through was added with 400 μL ethanol and transferred into a Zymo-Spin IIC Column to be centrifuged at 12.000 × g for 1 min. The DNA/RNA Prep buffer was added to the Zymo-Spin IIIC Column, previously transferred into a new Collection tube, and to the Zymo-Spin IIC Column. After two wash and centrifugation steps, DNase/RNase-Free Water was added to Zymo-Spin IIC Column and Zymo-Spin IIIC Column to extract RNA and DNA, respectively.

### Global 5-Methylcytosine DNA Analysis

The levels of 5-mC were measured by enzyme-linked immunosorbent assay (ELISA) method with the 5-methylcytosine DNA ELISA Kit (cat. #D5325, Zymo Research, Orange, CA, United States) using 100 ng extracted genomic DNA, according to manufacturers’ protocol. Briefly, extracted genomic DNA was first denatured at 98°C for 5 min in a thermal cycler, added to a 96 well plate and incubated at 37°C for 1 h for DNA coating. After washing, samples were blocked with 200 μL of 5-mC ELISA buffer and incubated at 37°C for 30 min. Antibody mix consisting of Anti-5-Methylcytosine (1:2,000) and secondary antibody (1:1,000) in 5-mC ELISA buffer was prepared, added to the plate and incubated at 37°C for 1 h. After washing, samples were incubated with horseradish peroxidase developer. Plates were read at 405 nm using a plate spectrophotometric reader (Genios Tecan, Männedorf, Switzerland). All samples were assayed in triplicate. To quantify 5-mC percentage in each DNA sample a standard curve was generated by preparing mixtures of the Negative Control (100 ng/μL) and Positive Control (100 ng/μL) to generate standards of known 5-mC percentage (see Table 1).

**TABLE 1:** Standard curve for % 5-mC determination.

(%) 5-mC	Negative control (100 ng (μL)	Positive control (100 ng (μL)
0	10.0	0
5	9.5	0.5
10	9.0	1.0
25	7.5	2.5
50	5.0	5.0
75	2.5	7.5
100	0	10.0

The absorbance for each mixture was plotted as a function of Absorbance 405 nm (*Y*-axis) vs. % 5-mC (*X*-axis). Using the equation below, derived from the logarithmic second order regression, the 5-mC percentage for DNA samples (unknowns) based on their absorbance was determined.

$$\%5mC=e^{\left\{\frac{\left(absorbance-y\mathrm{int}ercept\right)}{Slope}\right\}}$$

### Gene Expression Analysis by Real-Time qPCR

RNA integrity was checked by 1% agarose gel electrophoresis and concentrations were measured by using a Nanodrop 1,000 system spectrophotometer (Thermo Fisher Scientific, Waltham, MS, United States). RNA samples with absorbance 260/280 ratio >1.8 and <2.0 were subsequently reverse transcribed with the GeneAmp RNA PCR kit (Life Technologies, Carlsbad, CA, United States). Relative abundance of each mRNA of interest was assessed by real-time qRT-PCR using the Sybr Green gene expression Master Mix (Life Technologies, Carlsbad, CA, United States) in a Step One Real-Time PCR System (Life Technologies, Carlsbad, CA, United States) as previously described (Caputi et al., 2014). All data were normalized to glyceraldehyde-3-phosphate dehydrogenase (GAPDH) as the endogenous reference gene. Relative expression of different gene transcripts was calculated by the Delta-Delta Ct (DDCt) method and converted to relative expression ratio (2^−DDCt^) for statistical analysis (Livak and Schmittgen 2001). Primers used for PCR amplification were designed using Primer 3 and are reported in Supplementary Table S1 (see Supplementary material). Results are presented as fold changes in mRNA levels with respect to T0 levels defined as baseline values.

### Statistical Analysis

In both global 5-methylcytosine DNA assessment and gene expression analysis, samples were analyzed in technical triplicates, which were averaged before statistical analysis. The data were analyzed using a repeated measures mixed model approach, with Time-point as a (fixed) treatment factor and Patient (the subject factor) as a random effect. Patient age, surgery duration, pain scores, fentanyl dose, and morphine dose were investigated as covariates and included in the model if significant. An additional blocking factor Experiment was also included in the model to account for plate-to-plate variability, as data were analyzed in different experimental sessions using a complete block design (Bate et al., 2017). This analysis was followed by planned comparisons of the predicted means to compare the means of the post-surgical time-points (T1, T2, T3, and T4) with the mean of the T0 time-point. Cytokine gene expression data were log-transformed in order to obtain a normal distribution, which is a requirement for parametric analysis. The within-patient covariances were modeled using a compound symmetric covariance structure. The correlations between the response variables were assessed using Pearson’s product moment correlation coefficient. The results were deemed as significant for *p* values lower than 0.05. The analyses were performed by using InvivoStat software (Clark et al., 2012).

## Results

From October 2016 to June 2017, eleven patients undergoing major breast surgery performed under general anesthesia, referring to the Anesthesiology unit of “Policlinico di Sant’Orsola” were enrolled to the present study, after verifying the inclusion and exclusion criteria and signing the informed consent. Participant characteristics, anesthesia and surgical time and opioid consumption are described in Table 2. The dose of intraoperative fentanyl administered is a function of surgical time. This has led to the administration of non-standardized dosages. One patient refused opioids in the postoperative period and three patients stopped morphine infusion in the first 24 h after surgery due to PONV.

**TABLE 2:** Anthropometrics and Clinical data related to the study population.

**Sex (F/M)**	**11/0**
Age (yrs)	49 (39–64)
Height (cm)	166 (160–172)
Weight (Kg)	69 (54–96)
BMI	25 (18–34)
Anesthesia Time (min)	312 (120–756)
Surgical Time (min)	259 (61–690)
Intraoperative Fentanyl (mcg)	372 (250–550)
NRS r end of surgery	2 (0–5)
NRS m end of surgery	3 (1–7)
NRS r 24 h	2 (0–6)
NRS m 24 h	2,5 (0–6)
NRS r 48 h	1 (0–2)
NRS m 48 h	2 (0–3)
PONV 24 h (y/n)	4/4
PONV 48 h (y/n)	1/7
Antiemetics 24 h (y/n)	1/7
Antiemetics 48 h (y/n)	1/7
Morphine Consumption 24 h (mg)	24 (3–43)
Morphine Consumption 48 h (mg)	38 (3–67)

### DNA Methylation

**FIGURE 1:**
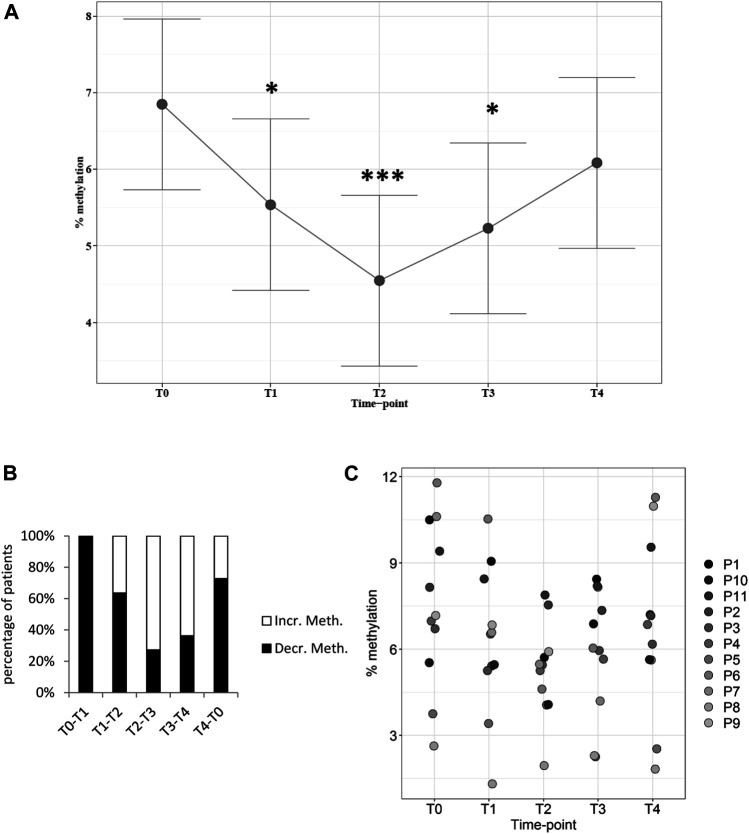
Global DNA methylation levels in patients at different time-points. **(A)**: Plot of the least square predicted means with 95% confidence intervals. The predicted least square means were calculated using a 1-way repeated measures mixed model. *p < 0.05; ***p < 0.001 in planned comparisons with T0 levels. **(B)**: variation in methylation levels between time-points. The percentage of patients showing increased (Incr. Meth.) or decreased (Decr. Meth.) methylation levels with respect to the previous time-point is shown. **(C)**: DNA methylation levels in each patient at every time-point. *n* = 11 patients repeatedly assessed at 5 time-points.

Global DNA methylation levels were assessed in each patient at five time-points. Samples were collected before surgery at the T0 time-point whereas T1 to T4 samples were collected at different hours after surgery (see Methods 2.3). The data were analyzed with a repeated measures mixed model approach, which showed a statistically significant effect with respect to the time-point as treatment factor (*p* = 0.0064). Methylation levels were then compared with T0 values, which defined the baseline for each individual patient. Planned comparisons of the predicted means comparing methylation levels at each time-point with the baseline T0 value showed a statistically significant reduction at time-points T1 (−18%, *p* = 0.036), T2 (−26%, *p* = 0.0005), and T3 (−18%, *p* = 0.0103), returning towards baseline at T4, which corresponded to 48 h after surgery (Figure 1A, see also Supplementary Figure S1 in Supplementary material). Methylation levels decreased between T0 and T1 in most patients and the decrease continued between T1 and T2 (Figure 1B). After this time-point, DNA methylation levels inverted the direction of change in most patients, pointing towards baseline levels, although they were not entirely regained at T4 (Figures 1B,C).

### mRNA Expression of DNMTs

**FIGURE 2:**
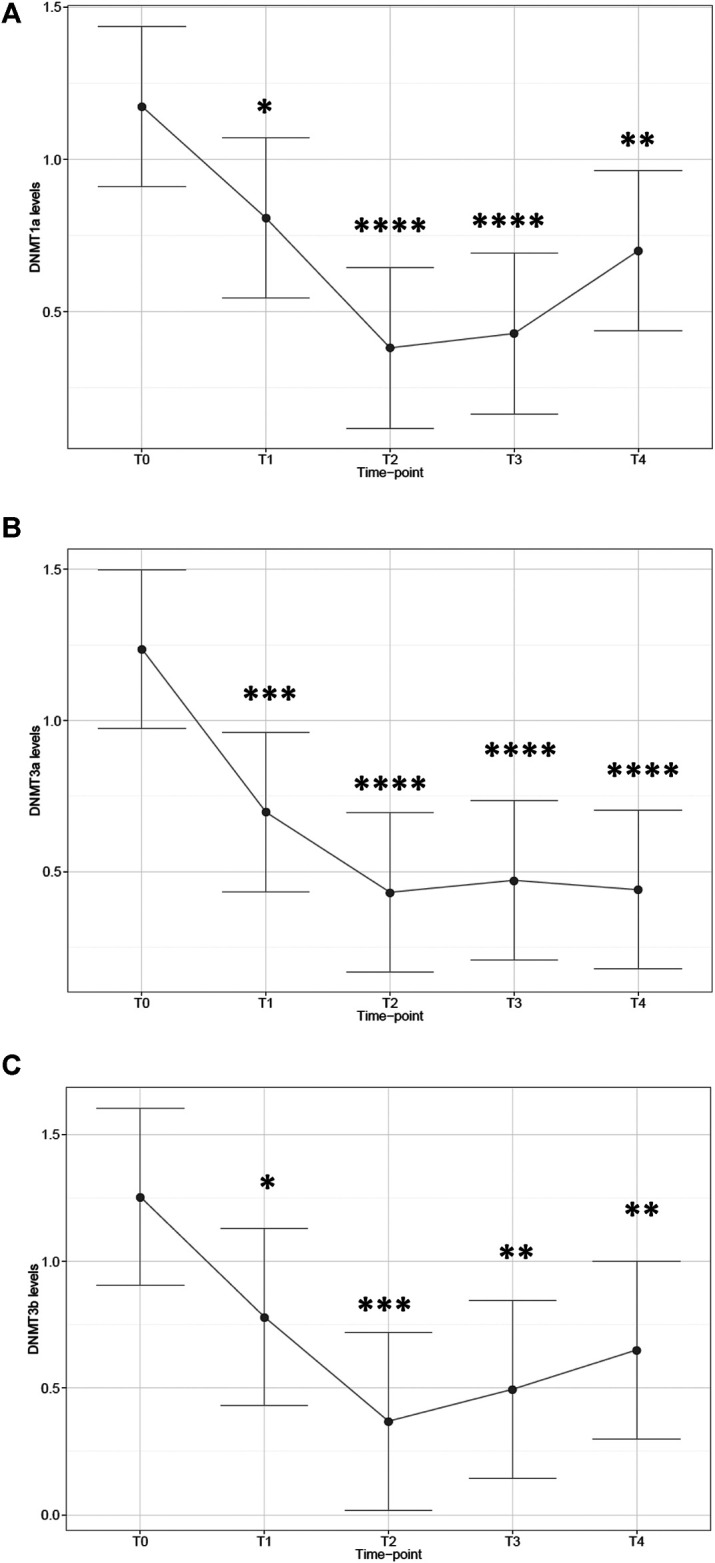
mRNA expression of DNA methyltransferase isoforms in patients at different time-points. Plot of the least square predicted means with 95% confidence intervals. The predicted least square means were calculated using a 1-way repeated measures mixed model. **(A)**: DNMT1a; **(B)**: DNMT3a; **(C)**: DNMT3b. *p < 0.05; **p < 0.01; ***p < 0.001; ****p < 0.0001 in planned comparisons with T0 levels. *n* = 11 patients repeatedly assessed at 5 time-points.

Next, we aimed to investigate whether the observed altered patterns of global DNA methylation were associated with modifications of DNMT gene expression. Therefore, we compared mRNA levels of the three most relevant DNMT isoforms at each time-point. Remarkably, we discovered that all three isoforms engendered a similar pattern of gene expression regulation. Specifically, DNMT1a mRNA expression was significantly altered by time-point (*p* < 0.0001), with statistically significant reductions in comparison with T0 levels (T1: −31%, *p* = 0.022; T2: −68%, *p* < 0.0001; T3: −64%, *p* < 0.0001; T4: −40%, *p* = 0.0037; Figure 2A). Likewise, DNMT3a showed significant modulation by time-point (*p* < 0.0001), with all post-surgical time-points displaying lower levels when compared with T0 (T1: −44%, *p* = 0.0009; T2: −65%, *p* < 0.0001; T3: −62%, *p* < 0.0001; T4: −64%, *p* < 0.0001; Figure 2B). On the same line, DNMT3b mRNA levels changed by time-point (*p* = 0.0021), with decreasing levels with respect to T0 which reached a trough at T2 (T1: −38%, *p* = 0.033; T2: −71%, *p* = 0.0002; T3: −61%, *p* = 0.001; T4: −48%, *p* = 0.0076; Figure 2C). For further details see also Supplementary Figures S2A,B,C in Supplementary material).

### Inflammatory Cytokine Expression

**FIGURE 3:**
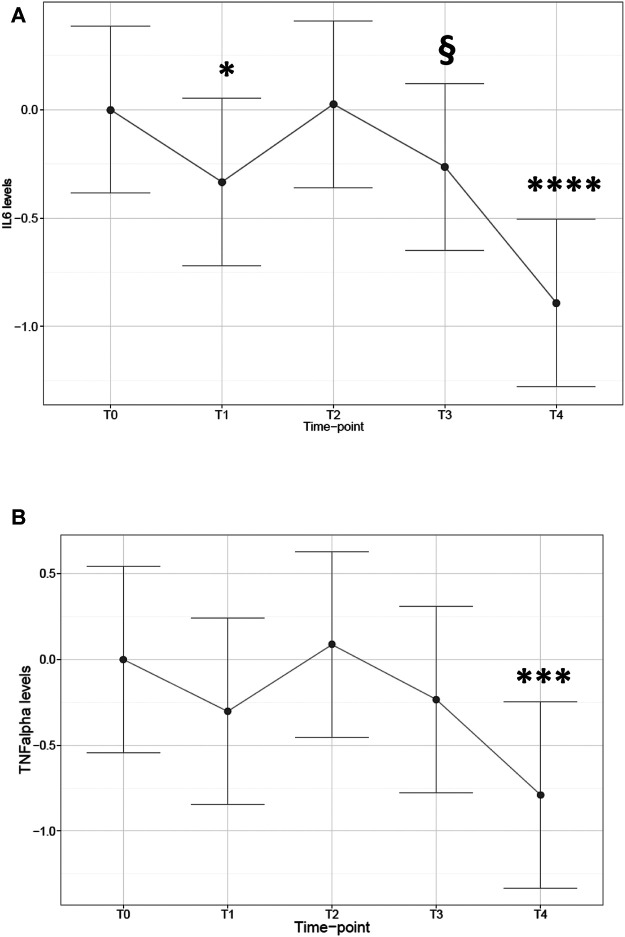
Cytokine mRNA expression in patients at different time-points. Plot of the least square predicted means with 95% confidence intervals. The predicted least square means were calculated using a 1-way repeated measures mixed model. **(A)**: IL6; **(B)**: TNFα. Ordinate axis is displayed on the Log10 scale. *p < 0.05; ***p < 0.001; ****p < 0.0001; ^§^0.05 < *p*< 0.1 in planned comparisons with T0 levels. *n* = 11 patients repeatedly assessed at 5 time-points.

Subsequently, we determined the expression patterns of the inflammatory cytokines IL6 and TNFα to investigate a possible inflammatory reaction to surgical stress. Cytokine expression showed a trend for increased expression at early time-points to end with a significant decrease at 48 h after surgery. In particular, a significant impact was observed on IL6 levels by time-point (*p* < 0.0001). Levels showed shifting directions during time (with respect to T0 baseline, −68% decrease at T1, *p* = 0.031; recovery at T2: +18%, *p* = n.s.; trend for decrease at T3, −52% *p* = 0.08; significant decrease at T4, −86% *p* < 0.0001; Figure 3A). A partially similar trend was observed for TNFα, which was modified by time-point (*p* = 0.0019), but cytokine levels compared with T0 baseline were significantly reduced only at T4 (−82%, *p* = 0.0007; Figure 3B). For further details see also Supplementary Figures S3A,B in Supplementary material).

### Correlations Between Changes in Global DNA Methylation and Changes in Gene Expression

We then investigated whether correlations existed between changes in methylation percentage and changes in the expression of DNMTs and cytokines. The aim of this analysis was to examine whether the parallel reduction of percentage methylation and DNMT levels observed from T0 to T2, followed by recovery from T2 to T4 correlated with each other. We also meant to explore if cytokine alterations displayed correlations with each other and with global methylation levels. To reach this objective, we calculated the difference of methylation levels and of DNMT or cytokine expression between T2 and T0 and between T4 and T2 and performed a correlation analysis between these variables. We discovered that changes in global methylation percentages were significantly correlated with expression variations of all DNMT isoforms (Table 3). Moreover, significant positive correlations were detected between variations of DNMT isoform expression, which were particularly strong between DNMT3a and DNMT3b (Table 3). We also discovered that cytokine changes showed highly significant correlations with each other (Table 3). In addition, IL6 changes showed a significant negative correlation with global methylation levels (Table 3). The identification of several significant correlations between DNMT isoforms and between DNMTs and cytokines, support the notion of a common regulatory mechanism on their coordinated expression.

**TABLE 3:** Correlation analysis between DNA methylation and DNMT and cytokine expression. Significant *p* values are displayed in bold. 0.05 < *p*< 0.1 values are displayed in italics.

Categorisation factor	First variable		Second variable	n	Correlation coefficient	Test statistic	*p*-value
T0	DNMT1a	vs.	DNMT3a	11	0.373	1.205	0.259
T0	DNMT1a	vs.	DNMT3b	11	0.262	0.814	0.4365
T0	DNMT1a	vs.	IL6	11	−0.272	−0.849	0.4179
T0	DNMT1a	vs.	percent meth.	11	0.549	1.972	*0.08*
T0	DNMT1a	vs.	TNFα	11	−0.333	−1.058	0.3175
T0	DNMT3a	vs.	DNMT3b	11	0.393	1.284	0.2312
T0	DNMT3a	vs.	IL6	11	0.191	0.585	0.5732
T0	DNMT3a	vs.	percent meth.	11	0.591	2.2	*0.0553*
T0	DNMT3a	vs.	TNFα	11	0.082	0.246	0.8109
T0	DNMT3b	vs.	IL6	11	−0.314	−0.991	0.3477
T0	DNMT3b	vs.	percent meth.	11	0.358	1.149	0.2804
T0	DNMT3b	vs.	TNFα	11	−0.441	−1.474	0.1745
T0	IL6	vs.	percent meth.	11	−0.066	−0.2	0.8462
T0	IL6	vs.	TNFα	11	0.986	17.536	**<0.0001**
T0	percent meth.	vs.	TNFα	11	−0.144	−0.437	0.6722
T1	DNMT1a	vs.	DNMT3a	11	0.331	1.052	0.3201
T1	DNMT1a	vs.	DNMT3b	11	0.603	2.266	**0.0496**
T1	DNMT1a	vs.	IL6	11	0.062	0.185	0.8571
T1	DNMT1a	vs.	percent meth.	11	0.164	0.5	0.629
T1	DNMT1a	vs.	TNFα	11	−0.054	−0.163	0.874
T1	DNMT3a	vs.	DNMT3b	11	−0.252	−0.783	0.4539
T1	DNMT3a	vs.	IL6	11	0.654	2.593	**0.0291**
T1	DNMT3a	vs.	percent meth.	11	0.005	0.014	0.9888
T1	DNMT3a	vs.	TNFα	11	0.449	1.508	0.1659
T1	DNMT3b	vs.	IL6	11	−0.651	−2.571	**0.0302**
T1	DNMT3b	vs.	percent meth.	11	−0.013	−0.039	0.97
T1	DNMT3b	vs.	TNFα	11	−0.63	−2.431	**0.0379**
T1	IL6	vs.	percent meth.	11	−0.172	−0.524	0.6131
T1	IL6	vs.	TNFα	11	0.85	4.847	**0.0009**
T1	percent meth.	vs.	TNFα	11	0.145	0.439	0.6708
T2	DNMT1a	vs.	DNMT3a	11	0.26	0.807	0.4403
T2	DNMT1a	vs.	DNMT3b	11	0.864	5.15	**0.0006**
T2	DNMT1a	vs.	IL6	11	−0.633	−2.454	**0.0365**
T2	DNMT1a	vs.	percent meth.	11	0.093	0.281	0.7852
T2	DNMT1a	vs.	TNFα	11	−0.625	−2.401	**0.0398**
T2	DNMT3a	vs.	DNMT3b	11	0.539	1.921	*0.0869*
T2	DNMT3a	vs.	IL6	11	−0.176	−0.535	0.6055
T2	DNMT3a	vs.	percent meth.	11	0.075	0.225	0.827
T2	DNMT3a	vs.	TNFα	11	−0.383	−1.243	0.2453
T2	DNMT3b	vs.	IL6	11	−0.759	−3.498	**0.0067**
T2	DNMT3b	vs.	percent meth.	11	0.325	1.03	0.3298
T2	DNMT3b	vs.	TNFα	11	−0.776	−3.694	**0.005**
T2	IL6	vs.	percent meth.	11	−0.391	−1.275	0.2341
T2	IL6	vs.	TNFα	11	0.861	5.075	**0.0007**
T2	percent meth.	vs.	TNFα	11	−0.5	−1.733	0.1171
T3	DNMT1a	vs.	DNMT3a	11	0.005	0.014	0.9891
T3	DNMT1a	vs.	DNMT3b	11	0.535	1.9	*0.0898*
T3	DNMT1a	vs.	IL6	11	0.114	0.345	0.7377
T3	DNMT1a	vs.	percent meth.	11	−0.38	−1.234	0.2484
T3	DNMT1a	vs.	TNFα	11	−0.033	−0.099	0.9234
T3	DNMT3a	vs.	DNMT3b	11	0.696	2.91	**0.0173**
T3	DNMT3a	vs.	IL6	11	0.605	2.28	0.0486
T3	DNMT3a	vs.	percent meth.	11	−0.226	−0.695	0.5047
T3	DNMT3a	vs.	TNFα	11	0.419	1.383	0.2
T3	DNMT3b	vs.	IL6	11	0.296	0.928	0.3776
T3	DNMT3b	vs.	percent meth.	11	−0.346	−1.105	0.2978
T3	DNMT3b	vs.	TNFα	11	0.135	0.407	0.6933
T3	IL6	vs.	percent meth.	11	−0.745	−3.349	**0.0085**
T3	IL6	vs.	TNFα	11	0.925	7.283	**<0.0001**
T3	percent meth.	vs.	TNFα	11	−0.772	−3.64	**0.0054**
T4	DNMT1a	vs.	DNMT3a	11	0.004	0.011	0.9912
T4	DNMT1a	vs.	DNMT3b	11	0.369	1.191	0.2642
T4	DNMT1a	vs.	IL6	11	−0.22	−0.677	0.5153
T4	DNMT1a	vs.	percent meth.	11	−0.353	−1.133	0.2865
T4	DNMT1a	vs.	TNFα	11	−0.249	−0.772	0.4597
T4	DNMT3a	vs.	DNMT3b	11	0.656	2.61	**0.0283**
T4	DNMT3a	vs.	IL6	11	0.347	1.109	0.296
T4	DNMT3a	vs.	percent meth.	11	0.191	0.582	0.5746
T4	DNMT3a	vs.	TNFα	11	0.114	0.344	0.7389
T4	DNMT3b	vs.	IL6	11	0.107	0.324	0.7533
T4	DNMT3b	vs.	percent meth.	11	0.168	0.511	0.6218
T4	DNMT3b	vs.	TNFα	11	−0.017	−0.051	0.9605
T4	IL6	vs.	percent meth.	11	−0.085	−0.257	0.8031
T4	IL6	vs.	TNFα	11	0.954	9.514	**<0.0001**
T4	percent meth.	vs.	TNFα	11	−0.131	−0.395	0.7017

## Discussion

Intraoperative analgesia during major surgery conducted under general anesthesia is based on the use of opiates and fentanyl is the most commonly employed. Evidence suggests that acute exposure to opiates may lead to OIH, which has been linked to epigenetic mechanisms, particularly to DNA methylation. Opiates are recognized as able to increase global DNA methylation levels (Doehring et al., 2013) in contrast to other pain medications such as local anesthetics (Lirk et al., 2015), although conflicting findings have been reported as well (Fragou et al., 2013).

Despite data available in scientific literature, which guided our primary hypothesis, this exploratory study has surprisingly shown that in patients undergoing general anesthesia with fentanyl administration for major breast surgery, global DNA methylation levels during the surgery were significantly lower compared to baseline. Methylation levels decreased in almost all patients immediately after the surgical incision and the decrease continued until the end of the surgery. In the postoperative period DNA methylation levels reversed the direction of change in most patients, pointing towards baseline levels.

The analysis of DNMT expression showed a massive decrease which paralleled the reduction of global DNA methylation. This finding is keeping with the crucial role played by DNMTs in regulating DNA methylation levels (Jurkowska et al., 2011). Moreover, the correspondence between reduced methylation levels and decreased DNMT expression, which were independently measured with different technological approaches, provided a confirm that an authentic and coherent biological response had been observed.

Widespread demethylation is a physiological event that occurs during development; however, hypomethylation also plays a role in the pathogenesis of cancer and other pathologies (Cedar and Bergman 2012). Reduced global methylation is observed during aging (Langevin et al., 2011) as well as in the response to environmental stress (Bollati et al., 2007; Baccarelli et al., 2009). Furthermore, in animal models of sciatic nerve chronic constriction injury - induced neuropathy, the intrathecal injection of 5-azacitidine, a DNMTs inhibitor, led to neuropathic pain reduction (Wang et al., 2011), thus highlighting an analgesic action associated with hypomethylation.

We hypothesize that the considerable DNA hypomethylation detected in our study during the intraoperative period may be related to the response of the body to surgical stress.

Hypomethylation is associated to increased probability of gene transcription, partly due to a modified chromatin packaging which facilitates binding of the transcriptional machinery. In this view, our results suggest that during the surgical procedure an increased transcription of a pool of genes involved in homeostasis regulation could have occurred. Indeed, patients undergoing major surgery revealed profound changes in homeostasis processes, mainly due to surgical stress and to inflammatory events. It is therefore reasonable to assume that this enhanced transcriptional activation may be involved in the protective response to the surgical insult. The activation of inflammatory signal transduction pathways leads to alterations in the expression of genes useful to avoid harmful consequences generated by a condition of surgical stress response. The systemic inflammatory response following surgery promotes healing and restores homeostasis in the body (Lin et al., 2000).

Cytokines are key mediators in this process and the response of TNFα and IL- 6 have been described in the context of surgical injury (Lin and Lowry, 1999). Indeed, TNFα and IL-6 represent two crucial mediators of the acute inflammatory response and both play a direct role in the onset and maintenance of pain (Latremoliere and Woolf, 2009; Sun et al., 2017).

In the present study cytokine expression showed a trend to increase at early time-points, which is remarkable since the potent anti-inflammatory agent dexamethasone had been administered prior to anesthesia induction in order to prevent PONV. Conversely, the significant decrease of these levels evaluated in our data at 48 h after surgery could be related to the compensatory capacities of the host designed to minimize the persistence of excessive inflammatory response. Despite the small number of patients enrolled in the study, it is noteworthy that cytokine mRNA levels showed significant correlations with methylation percentage and DNMT gene expression. Previous investigations reported an association between inflammatory responses and DNA hypomethylation, although the number of studies is too low to allow drawing definite conclusions (Gonzalez-Jaramillo et al., 2019). Moreover, the exposure to acute stress even of a moderate extent or at early times has been reported to be able to induce global patterns of DNA hypomethylation (Anier et al., 2014; Rodrigues et al., 2015).

The experimental design adopted in this exploratory study does not allow discriminating whether the occurrence of global hypomethylation was due to the administration of the general anesthetic agents utilized in this study. To our knowledge, no previous data are available to confirm or disprove such possibility. For example, the administration of local anesthetics has been described to be able to reduce methylation levels (Lirk et al., 2015). Therefore, it cannot be excluded that a similar effect is caused by general anesthetics, although the mechanism of action of these two classes of medications is not the same. Furthermore, it cannot be excluded that morphine administration, which was received by most patients starting from T2, could have contributed to restoring basal global DNA methylation levels after the decrease detected at T1 and T2. This observation could also be in agreement with studies showing hypermethylation during chronic opioid treatment (Doehring et al., 2013).

Limitations of this study are the small number of patients and the lack of correction for multiple comparisons. Further studies are needed to establish the role of intraoperative analgesia in the epigenetic modifications in a clinical context dominated by the stress response. In particular, future studies should assess: 1) the potential implications of the changes of DNA methylation between the intraoperative period, the end of the surgery and 48 h after surgery; 2) global DNA methylation changes in a period of time after T4 (48 h); 3) the correlation of DNA methylation with the opiates used (namely morphine); 4) the comparison of epigenetic responses in relation to different analgesic approaches (e.g. systemic opioids vs. regional anesthesia), and 5) site-specific DNA methylation levels on the promoter of selected genes, in order to identify the cellular pathways involved.

According to our findings and opposite to our initial hypothesis, we can argue that an overall DNA methylation level alteration cannot be merely explained by fentanyl administration, but rather by a complex clinical condition that significantly involves inflammatory responses to surgical stress. At this regard, it is interesting to point out the recent paper by Sadahiro and colleagues, who investigated the effects induced by major surgery on DNA methylation associated with immunological response to the stressful condition (Sadahiro et al., 2020).

Despite the possible limitation of our study, because of the measurement of the global methylation, our results agree with the above cited paper, showing alterations in DNA methylation at different time points, as well as in proinflammatory cytochines, after surgery.

In conclusion, the present study revealed DNA methylation variations in patients undergoing major surgery who were intraoperatively exposed to fentanyl; however, the alterations of global DNA methylation here observed seem to be more related to the surgical stress than to opiate exposure. Further studies focusing on post-translational mechanisms that would modulate inflammatory proteins during and after surgery, will be necessary to better clarify these phenomena.

## Data Availability

The original contributions presented in the study are included in the article/Supplementary Material, further inquiries can be directed to the corresponding author.
