# Supplementary material for: An Exploratory Pilot Study of Changes in Global DNA Methylation in Patients Undergoing Major Breast Surgery Under Opioid-Based General Anesthesia
Source: Front Pharmacol. 2021 Sep 21;12:733577. doi: 10.3389/fphar.2021.733577 (PMC8491974; doi:10.3389/fphar.2021.733577)
Supplement: Supplementary file 1 [file Table1.docx]

Table S1. Primer sequences used for real-time qPCR.

|  | **Forward (5’- 3’)** | **Reverse (5’- 3’)** |
| --- | --- | --- |
| **DNMT1** | CCCCTGAGCCCTACCGAAT | CTCGCTGGAGTGGACTTGTG |
| **DNMT3a** | TATTGATGAGCGCACAAGAGAGC | GGGTGTTCCAGGGTAACATTGAG |
| **DNMT3b** | GGCAAGTTCTCCGAGGTCTCT | TGGTACATGGCTTTTCGATAGGA |
| **IL-6** | AATGAGGAGACTTGCCTGGT | GCAGGAACTGGATCAGGACT |
| **TNFα** | TTCATGAAGCTCTCACTTCTC | CCACAGTGGGTAGGAGAATG |
| **GAPDH** | GGTCGGAGTCAACGGATTT | TGGACTCCACGACGTACTCA |
