# Supplementary material for: An Exploratory Pilot Study of Changes in Global DNA Methylation in Patients Undergoing Major Breast Surgery Under Opioid-Based General Anesthesia
Source: Front Pharmacol. 2021 Sep 21;12:733577. doi: 10.3389/fphar.2021.733577 (PMC8491974; doi:10.3389/fphar.2021.733577)
Supplement: Supplementary file 2 [file DataSheet1.pdf]

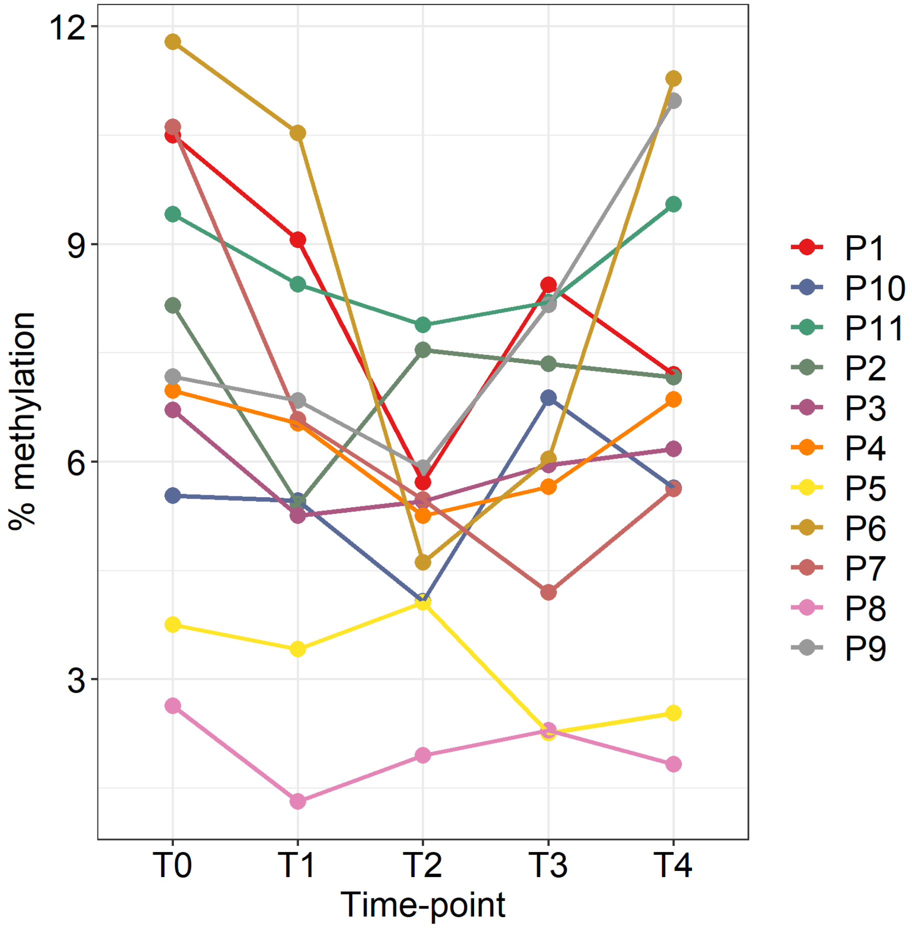

Supplementary Figure S1: Percentage of global DNA methylation, case profiles plot of the raw data for each patient at each time-point.

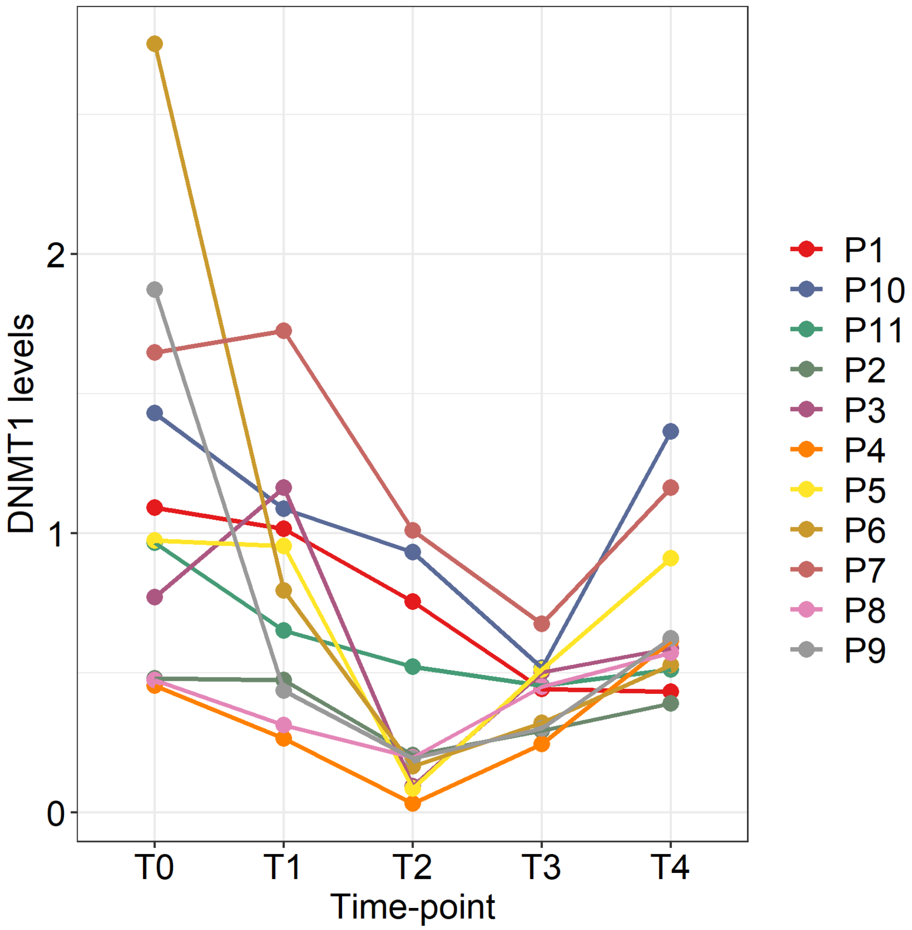

Supplementary Figure S2A: Relative DNMT1 mRNA expression, case profiles plot of the raw data for each patient at each time-point.

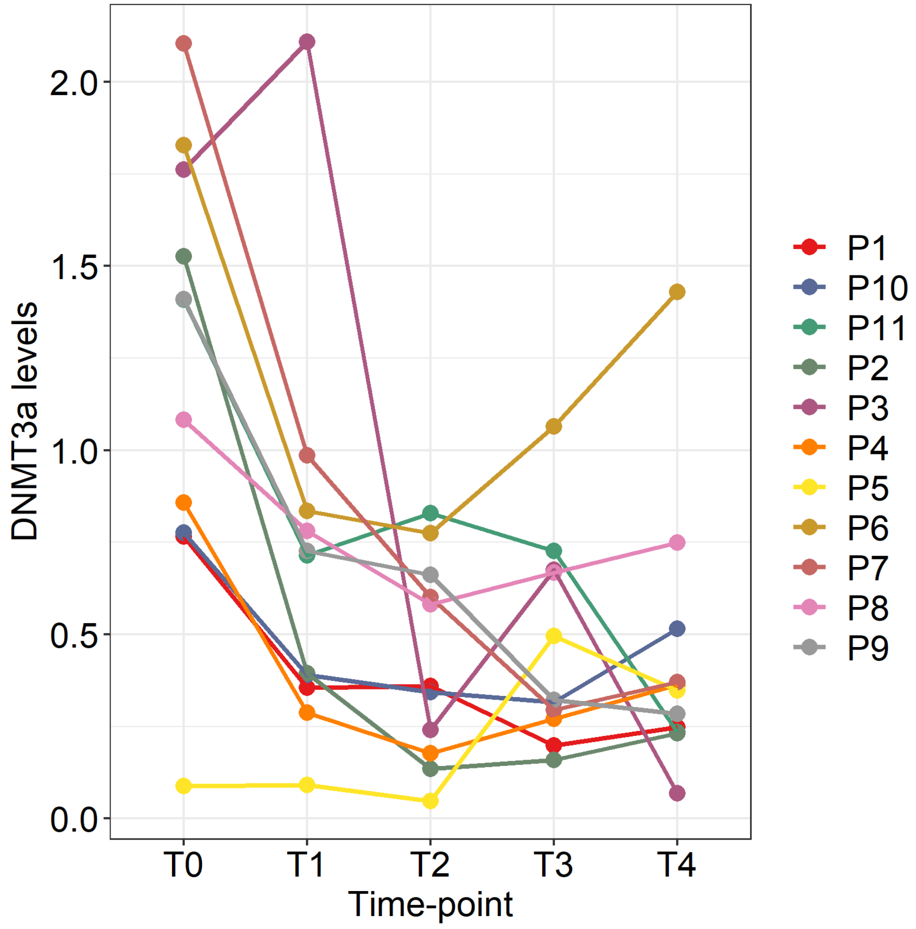

Supplementary Figure S2B: Relative DNMT3a mRNA expression, case profiles plot of the raw data for each patient at each time-point.

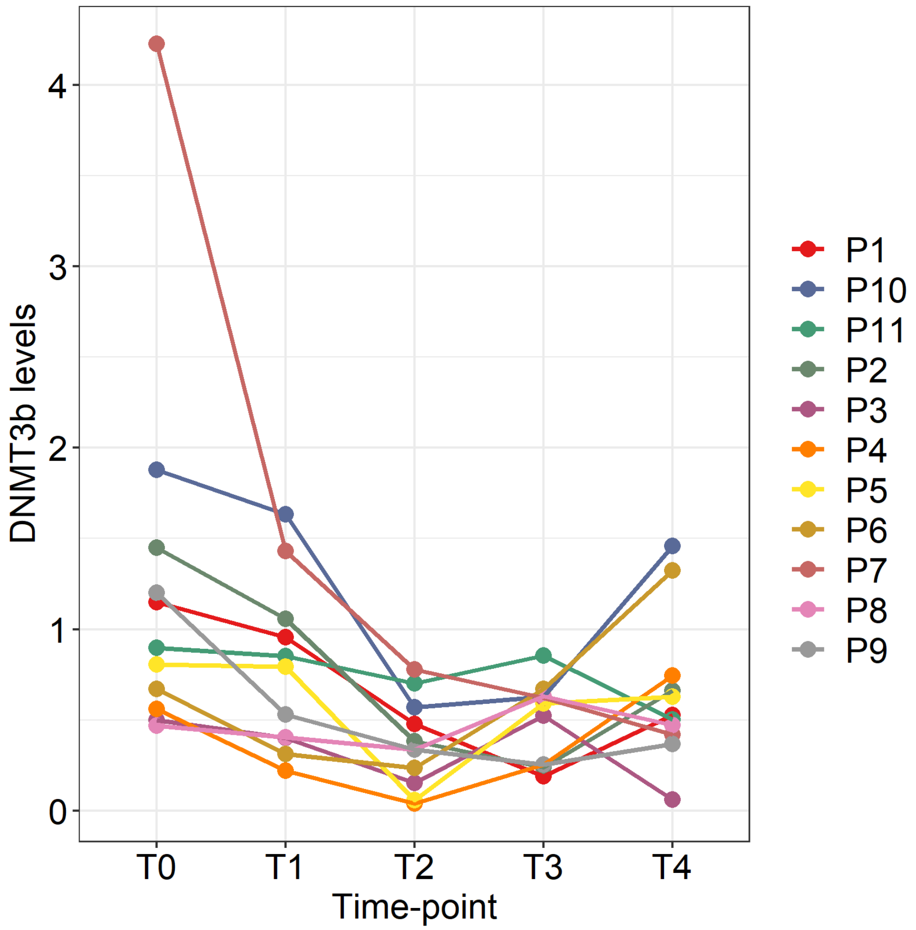

Supplementary Figure S2C: Relative DNMT3b mRNA expression, case profiles plot of the raw data for each patient at each time-point.

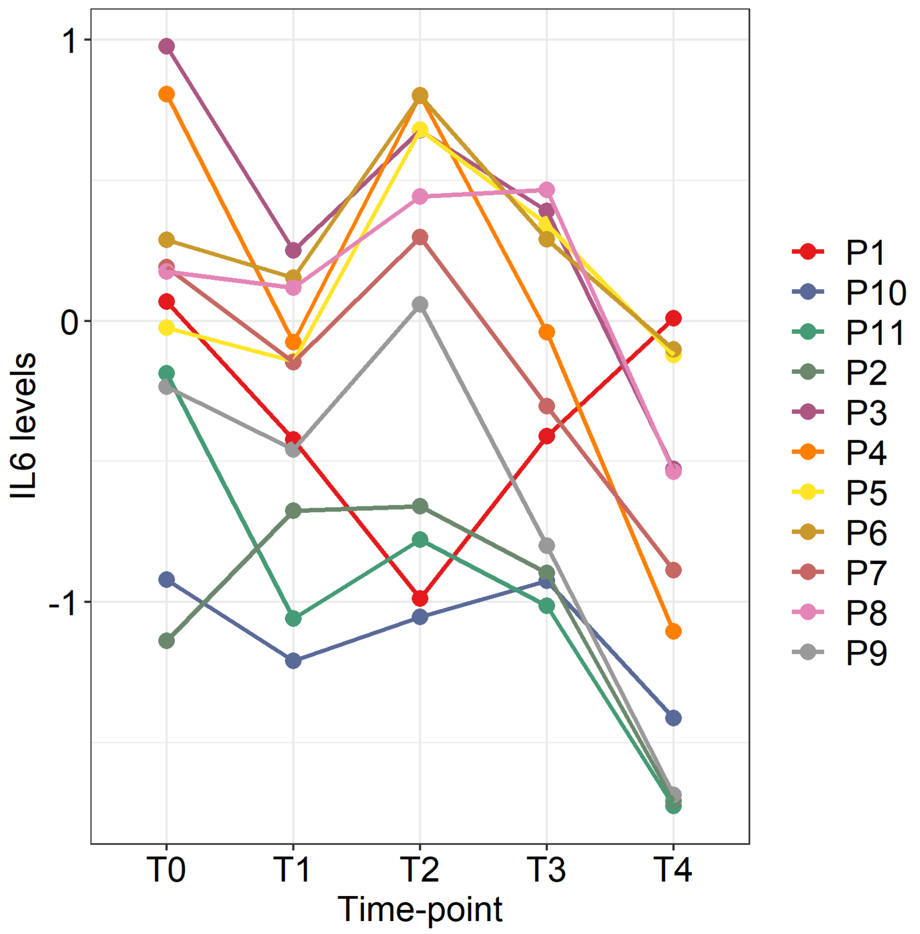

Supplementary Figure S3A: Relative IL6 mRNA expression, case profiles plot of the raw data for each patient at each time-point. Y axis is on the Log10 scale.

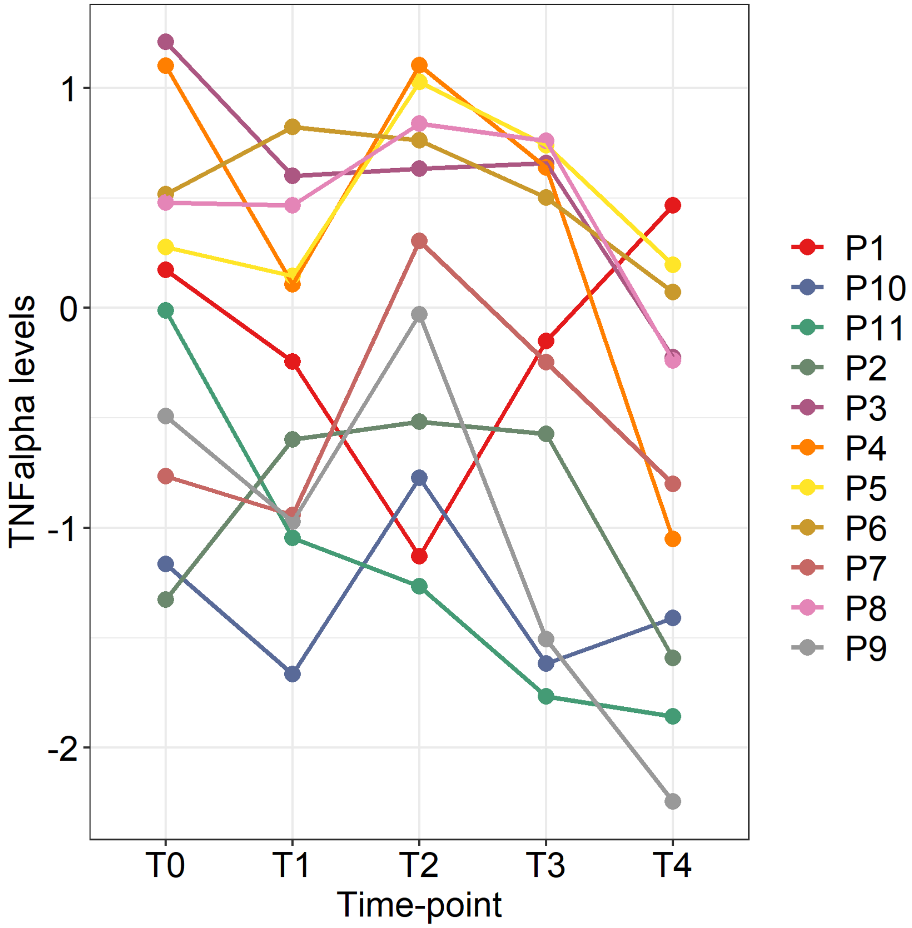

Supplementary Figure S3B: Relative TNFα mRNA expression, case profiles plot of the raw data for each patient at each time-point. Y axis is on the Log10 scale.
